# Coding-complete genome of bluetongue virus serotype 3 isolated in 2015 from dairy cattle in Colorado

**DOI:** 10.1128/mra.01280-24

**Published:** 2025-09-02

**Authors:** Jennifer Kopanke, Justin S. Lee, Teresa M. Garcia, Tyler J. Sherman, Mollie Burton, Molly Carpenter, Tillie Dunham, Christie E. Mayo

**Affiliations:** 1 Department of Comparative Medicine, Oregon Health & Science University 6684, Portland, Oregon, USA; 2 Department of Microbiology, Immunology, and Pathology, Colorado State University 164597 , Fort Collins, Colorado, USA; 3 Veterinary Diagnostic Services, New Mexico Department of Agriculture, Albuquerque, New Mexico, USA; 4 Diagnostic Medicine Center, Colorado State University 3447, Fort Collins, Colorado, USA; Katholieke Universiteit Leuven, Leuven, Belgium

**Keywords:** bluetongue virus

## Abstract

Bluetongue is a reemerging disease of agricultural livestock. Sporadic outbreaks occur endemically, and the introduction of non-endemic strains is common. Reassortment of geographically dispersed strains may produce pathogenic variants; however, virulence determinants remain unclear. We present the complete coding genome of a strain of bluetongue virus serotype 3 previously collected under active surveillance.

## ANNOUNCEMENT

Bluetongue virus (BTV) is the prototype *Orbivirus* (family *Sedoreoviridae*) and has caused sporadic outbreaks of disease in ruminants on nearly all continents since its first characterization in 1876 (1, 2). Genomic determinants of virulence remain incompletely understood; however, reassortment between strains is common and may yield phenotypically novel variants (3). While bluetongue virus serotype 3 (BTV-3) is considered an established serotype in the United States as of September 2022, a novel BTV-3 strain is currently causing ongoing outbreaks in Europe (4–6). BTV-3 was first detected in the United States in Florida in 1999 and has since been detected in Mississippi, Arkansas, South Dakota, and Texas, suggestive of regional dissemination over the last few decades (6).

Samples sequenced during a graduate study in 2015 were retrospectively analyzed. Briefly, BTV was isolated from quantitative PCR-positive whole blood on bovine pulmonary artery endothelial cells maintained as previously described (7). The sample described here was isolated from a non-clinical bovine born and raised on a dairy farm in Colorado. Nucleic acids were extracted from cell culture supernatant using the MagMAX Pathogen RNA/DNA Kit (Thermo Fisher). Samples were treated with TURBO DNase (Thermo Fisher) and incubated with LiCl to precipitate single-stranded RNA as previously described (8). A final 1.25× clean-up was performed using the MagMAX Pathogen RNA/DNA Kit. Libraries were prepared for whole-genome sequencing using the ScriptSeq (v.2) RNA-seq library kit (Illumina) with an initial fragmentation time of 90 seconds at 80°C. Fragments were size-selected on a 1% agarose gel (300–800 bp), purified using the QIAquick Gel Extraction kit (Qiagen), and screened for concentration and quality using a 2200 TapeStation (Agilent) and KAPA Library quantification (Kapa Biosystems) as specified by the manufacturer. Finally, sequencing took place on a NextSeq 500 using the mid-output 300 cycle NextSeq (v.2) reagents (Illumina). Default parameters were used to first remove low-quality reads and adapters from sequences using cutadapt (v.3.4) and bbduk (v.39.01). Duplicate reads were then collapsed using cd-hit-dup (v.4.8.1). We then performed repeat alignments using Bowtie2 (v.2.5.2) to find the best reference sequence available, classified by the most reads aligned for each segment. We found the following best references for segments 1–10, respectively (L20445, KY092160, AF017281, KF986515, NC_006025, KY092047, J04365, KY091991, AF403418, and AF044372), which were then used for downstream variant calling and consensus generation. Variant calling and consensus generation were performed using bcftools (v.1.9).

BLAST alignment of all BTV3/CO/2015 segments against available references in the National Center for Biotechnology Information detected homologous sequences most identical (>97.0%) to other BTV isolates circulating in the Southeastern United States, Central America, or the Caribbean (Table 1). When compared directly to the recently characterized BTV-3/NET2023 (OR603992–OR604001), isolated from *Ovis aries*, pairwise identity between individual segments was all <90.0% except for segment 7 (92.6%). Regular surveillance of BTV that includes characterization via whole-genome sequencing is crucial to our understanding of viral evolution patterns and detection of previously unreported serotypes.

**TABLE 1:** Summary of assembly metrics for BTV3/CO/2015 and most similar GenBank gene segment matches from field isolates^*b*^

Gene	Size (bp)	GC (%)	Mean depth of coverage (*x*)	Closest GenBank match (%)^*a*^	GenBank match serotype	GenBank match origin
VP1	3,910	42.2	134.14	KX164079 (100/98.8)	12	TX/2008/WTD
VP2	2,932	41.4	196.76	KY092160 (100/99.3)	3	FL/2001/WTD
VP3	2,731	43.1	144.47	KY092132 (100/98.9)	3	MS/2009/WTD
VP4	1,978	43.7	201.41	KY092115 (100/97.2)	18	GU/1990/UN
NS1	1,757	43.6	693.26	M97762 (100.98.0)	13	UN/1993/UN
VP5	1,630	43.4	613.61	KY092041 (100/99.5)	3	HO/1990/UN
VP7	1,130	48.0	2212.33	KY092024 (100/98.3)	3	MS/2009/WTD
NS2	1,106	44.7	1715.49	AY855289 (100/98.8)	13	FL/1999/OA
VP6	1,045	49.3	2476.29	KY091971 (100/98.8)	3	MS/2009/WTD
NS3	805	45.3	603.49	AF044379 (100/98.0)	10	CA/1980/BT

^*a*^ Query coverage/identity.

^*b*^ BT*, Bos taurus*; FL, Florida; GU, Guatemala; HO, Honduras; IN, India; MS, Mississippi; NS, non-structural protein; OA, *Ovis aires*; PR, Puerto Rico; TX, Texas; UN, unknown; VP, viral protein; WTD, white-tailed deer.

## Data Availability

The genome sequences described here have been deposited in GenBank under accession numbers PQ584054 through PQ584063. Raw sequencing reads are available in the Sequence Read Archive database under BioProject accession number PRJNA1209120.

## References

[1] Alkhamis MA, Aguilar-Vega C, Fountain-Jones NM, Lin K, Perez AM, Sánchez-Vizcaíno JM. 2020. Global emergence and evolutionary dynamics of bluetongue virus. Sci Rep 10:21677. doi:10.1038/s41598-020-78673-9 33303862 PMC7729867

[2] Matthijnssens J, Attoui H, Bányai K, Brussaard CPD, Danthi P, del Vas M, Dermody TS, Duncan R, Fāng Q, Johne R, Mertens PPC, Mohd Jaafar F, Patton JT, Sasaya T, Suzuki N, Wei T. 2022. ICTV virus taxonomy profile: Sedoreoviridae 2022. J Gen Virol 103. doi:10.1099/jgv.0.001781 PMC12643109 36215107

[3] Sanders C, Veronesi E, Rajko-Nenow P, Mertens PPC, Batten C, Gubbins S, Carpenter S, Darpel K. 2022. Field-reassortment of bluetongue virus illustrates plasticity of virus associated phenotypic traits in the arthropod vector and mammalian host in vivo. J Virol 96:e0053122. doi:10.1128/jvi.00531-22 35727032 PMC9278112

[4] USDA APHIS. 2024. Bluetongue. Cattle and Bison Diseases.

[5] van den Brink K, Santman-Berends I, Harkema L, Scherpenzeel CGM, Dijkstra E, Bisschop PIH, Peterson K, van de Burgwal NS, Waldeck HWF, Dijkstra T, Holwerda M, Spierenburg MAH, van den Brom R. 2024. Bluetongue virus serotype 3 in ruminants in the Netherlands: clinical signs, seroprevalence and pathological findings. Vet Rec 195:e4533. doi:10.1002/vetr.4533 39148262

[6] Holwerda M, Santman-Berends I, Harders F, Engelsma M, Vloet RPM, Dijkstra E, van Gennip RGP, Mars MH, Spierenburg M, Roos L, van den Brom R, van Rijn PA. 2024. Emergence of bluetongue virus serotype 3, the Netherlands, September 2023. Emerg Infect Dis 30:1552–1561. doi:10.3201/eid3008.231331 38941965 PMC11286052

[7] Kopanke JH, Lee JS, Stenglein MD, Mayo CE. 2020. The genetic diversification of a single bluetongue virus strain using an in vitro model of alternating-host transmission. Viruses 12:1038. doi:10.3390/v12091038 32961886 PMC7551957

[8] Kopanke J, Lee J, Stenglein M, Mayo C. 2021. In vitro reassortment between endemic bluetongue viruses features global shifts in segment frequencies and preferred segment combinations. Microorganisms 9:405. doi:10.3390/microorganisms9020405 33669284 PMC7920030

